# A case report of neurological adverse events caused by short-term and low-dose treatment of mitotane: The role of therapeutic drug monitoring

**DOI:** 10.1097/MD.0000000000022620

**Published:** 2020-10-02

**Authors:** Xin Liu, Qiang Fu, Yan Tang, Jian-hua Deng, Dan Mei, Bo Zhang

**Affiliations:** a Department of Pharmacy; b Department of Urology, Peking Union Medical College Hospital, Chinese Academy of Medical Sciences and Peking Union Medical College, Beijing, China.

**Keywords:** mitotane, plasma level, therapeutic drug monitoring, toxicity

## Abstract

**Rationale:**

Low-dose mitotane has been widely used for many decades in patients with advanced adrenocortical carcinoma (ACC), which exhibited good safety profiles compared with the high-dose regimen. The clinical efficacy and toxicity of mitotane are closely related to its plasma concentration, and therapeutic drug monitoring (TDM) is recommended. Until now, no severe adverse drug reaction (ADR) related to the toxic plasma level after a short-term treatment of low-dose mitotane has been published.

**Patient concerns:**

A 50-year-old Chinese female presented with severe neurological adverse events related to a toxic plasma levels of 42.8 mg/L after 4 months treatment of low-dose mitotane.

**Diagnoses:**

During the course of therapy, no other medication could cause neurological adverse events. Therefore, we suspected a high sensitivity to the side effect of mitotane related to a toxic plasma level.

**Interventions:**

Treatment of mitotane was stopped.

**Outcomes:**

The trough plasma concentration of mitotane decreased to 18.7 mg/mL after one and a half months, and the neurological symptoms gradually improved after drug discontinuance.

**Lessons:**

The present case provides the first report of severe neurological adverse events induced by the short-term use of low-dose mitotane for adjuvant treatment in a patient with ACC, indicating that potentially severe ADR can also occur when using low-dose regimen in the early stage of treatment. TDM and early recognition could result in a favorable outcome.

## 1 Introduction

Adrenocortical carcinoma (ACC) is a rare and aggressive solid tumor characterized by a 5-year survival rate of below 15% for metastatic disease.^[1]^ Mitotane (1,1-(*o*,*p*′-dichlorodiphenyl)-2,2-dichloroethane, *o*,*p*′-DDD) is recommended as the most common adjuvant therapy for patients with advanced ACC.^[2]^ It is the only antineoplastic agent for ACC specifically approved by the U.S. Food and Drug Administration, and the European Medicine Executive Agency recognized it as an orphan drug. The toxicity of mitotane represents a major limit to its suitability in the treatment of ACC patients, including hepatic disorder, leukopenia, neurologic symptoms and gastrointestinal disturbances.^[3]^

Clinical studies have demonstrated that the mitotane plasma trough concentrations within 14 to 20 mg/L are correlated with a higher response rate.[^4^,^5^,^6^] A significant increase in neurological toxicity and no further benefit in terms of efficiency have been reported when plasma mitotane levels exceed 20 mg/L. However, the onset of digestive toxicity was proved not to be related to the plasma mitotane level. In addition, mitotane plasma levels >30 mg/L are extremely rare, and it was suggested that the plasma level should not be exceeded this value until a better understanding of the clinical toxicity achieved.^[7]^ Several dosing regimens have been proposed to attain the target plasma ranges. Nevertheless, a significant number of patients fail to reach therapeutic plasma mitotane levels at all, for reasons not yet known.^[8]^ Low dose regimen begins with 3 g per day for 3 to 4 months then continue with 1 to 2 g per day is proved to be able to consistently provide elevated mitotane levels with well-tolerated toxicity, even though the time lag necessary for attaining mitotane levels greater than 14 mg/L was particularly long in some patients.^[9]^ A recent study indicated that achievement of target mitotane levels required a median time of 8 (5–19) months from the start of therapy in patients with a low-dose regimen, while plasma concentration of 10% patients never achieved levels above 14 mg/L.^[8]^ Otherwise, high dosing regimen consisting in progressively attaining 4 to 9 g per day within 2 weeks and maintaining this dose at least 6 weeks allow to reach the therapeutic range earlier, but it is considered more likely to provide serious side effects.[^10^,^11^]

Neurological adverse effects related to toxic plasma levels of mitotane had been reported in a few case reports, in which patients were treated either with high-dose regimen (6 and 4 g/d, respectively)[^12^,^13^] or a long-term treatment (1 year).^[14]^ Herein, we present the first known case of neurotoxicity related with a toxic plasma level of mitotane in a Chinese patient with 4 months of low-dose mitotane treatment, which indicates that the toxic plasma level and the related serious adverse effects can also occur in short-term and low-dose mitotane treatment and emphasizes the importance of the therapeutic drug monitoring (TDM) during mitotane treatment, even in the early stages.

## 2 Case presentation

A 50-year-old Chinese woman (BMI: 22.3) faced with the suspicion of ACC underwent surgical resection in April 2018. The following pathological analysis confirmed the diagnosis of malignant ACC with Ki-67 protein proliferation index of 20%. The patient regularly went to the hospital for review after the surgery, and a computerized tomography scan in December 2018 showed no signs of tumor recurrence. In May 2019, she referred to the hospital complained about left abdominal discomfort, and ^18^F-FDG PET revealed abnormal uptake of the radiotracer in the region of the left adrenal gland, which indicated the consideration of the tumor recurrence. Doctors consultations did not recommend surgery again. As per guidelines, a low dosing regimen of oral mitotane chemotherapy (2 g/d) started on July 21, 2019, and the treatment was associated with cortisol replacement therapy (hydrocortisone acetate, 30 mg/d) to maintain an adequate hemodynamic. After 2 weeks of treatment, adverse effects related to gastrointestinal symptoms (nausea, diarrhea, and anorexia) occurred. The plasma level of mitotane measured by high performance liquid chromatography (HPLC) was 5.85 mg/L (August 12, 2019), which had not yet reached the therapeutic range. Thus, the dose of mitotane was progressively increased to 3 g/d (August 15, 2019). Three months later (November 14, 2019), the patient presented neurological side effects including dizziness, fatigue, confusion, movement and coordination disorders, memory loss, concentration difficulty, and difficulty to talk, whereas nausea and diarrhea still persisted. Considering the good safety profile of hydrocortisone acetate according to the drug label and literature,[^15^,^16^] a neurotoxicity of mitotane was suspected, with degree II memory loss, coordination disorders, and degree I concentration difficulty, dizziness, language disability, and confusion, according to the CTCAE (Common Terminology Criteria for Adverse Events) V5.0. Concomitantly, results of therapeutic drug monitoring revealed a concentration of 42.8 mg/L, which confirmed a mitotane toxic concentration despite the patient with a short term and low-dose treatment. Mitotane was promptly discontinued, and the trough plasma concentration of mitotane decreased to 18.7 mg/L on December 26, 2019. At that time, neurological symptoms significantly improved, whereas nausea and diarrhea persisted.

## 3 Discussion

The rare disease is a public health issue worldwide, especially in China, the most populous country in the world. The most urgent problem for the treatment of rare diseases in China is the limited availability of the orphan drug. However, progress has been made now, and orphan drugs from foreign countries will be legally available.[^17^,^18^] Since 2018, 37 urgently needed orphan drugs qualified for accelerated and simplified approval by the National Medical Products Administration of China. In particular, our hospital (Peking Union Medical College Hospital) legally purchased mitotane tablets through the green channel approval process in 2019.

Mitotane was licensed as an orphan drug in Europe in 2004 for unresectable, relapsed or metastatic ACC. It has been recently indicated that the objective response of mitotane monotherapy is up to 20% of patients with advanced ACC, with progression-free survival and overall survival of 4.1 and 18.5 months, respectively.^[19]^ Although it has been used for a long time, its pharmacological properties and exact mechanism of action are still debated and remain to be more explored.[^20^,^21^] Previous studies indicated that mitotane has direct cytotoxic effects on the adrenal cortex inducing focal degeneration of the zona fasciculata and the zona reticularis.^[22]^ In addition, it exhibits tumor specificity as its adrenolytic effects seem to be enhanced by the presence of CYP11B activity in cortisol secreting tumors.^[23]^ The in vitro data also indicated that mitotane could alter mitochondrial respiratory chain activity by inducing cytochrome C oxidase defect in human ACC cells and inhibit Sterol-O-acyl transferase 1, inducing endoplasmic reticulum stress in ACC cells, in turn leading to apoptosis.^[24]^ Currently, no available pharmacological options are better than mitotane, and the efficacy of the drug depends on achieving therapeutic plasma levels of greater than or equal to 14 mg/L. However, a significant increase in neurological toxicity has been reported when plasma mitotane levels exceed 20 mg/L.^[25]^ Therefore, mitotane plasma monitoring is currently recommended for targeting and maintaining plasma levels between 14 and 20 mg/L.

Being a highly lipophilic drug, mitotane has a wide tissue distribution and a long terminal half-life, so that the steady-state may be achieved after a long-term of therapy. In fact, a high-dose regimen has been advocated to allow a rapid rise of plasma mitotane concentrations to avoid any delay in drug activity. However, toxicity associated with such a regimen is of concern.^[26]^ Toxic adverse reactions have been reported in patients with high-dose mitotane treatment,[^12^,^13^,^14^] and less attention has been paid to the treatment with low-dose mitotane, especially for the early phase of treatment. To the best of our knowledge, toxic plasma levels, and the related neurological toxicity of mitotane has not been previously reported. Our case was in agreement with the concept that individual differences in mitotane metabolism and other still unknown factors influence plasma concentrations, especially in the first phase of treatment.[^27^,^28^] In this case, the patient took only 2 medicines (hydrocortisone acetate and mitotane) before the neurological side effects. In consideration of the good safety profile of hydrocortisone acetate in long-term treatment and a toxic mitotane concentration (42.8 mg/L), we ruled out the contribution of hydrocortisone acetate. Although there are some limitations in this case report, such as the absence of pharmacogenetic studies, the present case calls for attention to the severe adverse effects and toxic plasma levels in the early stage of low-dose mitotane treatment and strengthens the value of mitotane TDM, not only for maximizing therapeutic efficacy but also for minimizing the incidence of severe toxicities. A close TDM should be carried out in the condition of neurological toxicity to evaluate the exposure of the patient to mitotane. Finally, the decision of mitotane discontinuation should be considered from the early signs of neurotoxicity or in the presence of elevated mitotane blood concentration in consideration of that mitotane presents a very long terminal half-life in vivo.

In conclusion, the present case provided the first severe neurological adverse and the related toxic plasma level after 4 months treatment of low-dose mitotane, which indicated that severe toxicities could also occur in the early phase of low-dose mitotane treatment. Our case demonstrates the usefulness of mitotane TDM in avoiding drug toxicity, even with the low-dose regimen in an early stage.

## Author contributions

**Conceptualization:** Bo Zhang.

**Data curation:** Xin Liu.

**Funding acquisition:** Xin Liu.

**Investigation:** Xin Liu.

**Methodology:** Qiang Fu.

**Resources:** Jian-hua Deng.

**Validation:** Dan Mei.

**Writing – original draft:** Xin Liu.

**Writing – review & editing:** Qiang Fu, Yan Tang, Bo Zhang.
